# Application of a color scanner for ^60^Co high dose rate brachytherapy dosimetry with EBT radiochromic film

**DOI:** 10.2478/v10019-012-0015-1

**Published:** 2012-11-09

**Authors:** Mahdi Ghorbani, Mohammad Taghi Bahreyni Toossi, Ali Asghar Mowlavi, Shahram Bayani Roodi, Ali Soleimani Meigooni

**Affiliations:** 1 North Khorasan University of Medical Sciences, Bojnurd, Iran; 2 Medical Physics Research Center, Medical Physics Department, Faculty of Medicine, Mashhad University of Medical Sciences, Mashhad, Iran; 3 Physics Department, School of Sciences, Hakim Sabzevari University, Sabzevar, Iran; 4 Comprehensive Cancer Center of Nevada, 3730 S. Eastern Avenue, Las Vegas, Nevada, USA

**Keywords:** color scanner, GZP6 brachytherapy source, ^60^Co high dose rate source, HDR, EBT radiochromic film

## Abstract

**Background.:**

The aim of this study is to evaluate the performance of a color scanner as a radiochromic film reader in two dimensional dosimetry around a high dose rate brachytherapy source.

**Materials and methods:**

A Microtek ScanMaker 1000XL film scanner was utilized for the measurement of dose distribution around a high dose rate GZP6 ^60^Co brachytherapy source with GafChromic® EBT radiochromic films. In these investigations, the non-uniformity of the film and scanner response, combined, as well as the films sensitivity to scanner’s light source was evaluated using multiple samples of films, prior to the source dosimetry. The results of these measurements were compared with the Monte Carlo simulated data using MCNPX code. In addition, isodose curves acquired by radiochromic films and Monte Carlo simulation were compared with those provided by the GZP6 treatment planning system.

**Results:**

Scanning of samples of uniformly irradiated films demonstrated approximately 2.85% and 4.97% nonuniformity of the response, respectively in the longitudinal and transverse directions of the film. Our findings have also indicated that the film response is not affected by the exposure to the scanner’s light source, particularly in multiple scanning of film. The results of radiochromic film measurements are in good agreement with the Monte Carlo calculations (4%) and the corresponding dose values presented by the GZP6 treatment planning system (5%).

**Conclusions:**

The results of these investigations indicate that the Microtek ScanMaker 1000XL color scanner in conjunction with GafChromic EBT film is a reliable system for dosimetric evaluation of a high dose rate brachytherapy source.

## Introduction

Radiation dosimetry is essential for quality assurance in diagnostic and therapeutic radiology.^1^,^2^ One of the most frequent tools for dosimetry are radiochromic films (RCF) because (1) they have nearly a tissue equivalent base material, (2) their spatial resolution is high, (3) they have a minor energy and dose rate dependency, and (4) they do not require a darkroom and developing.^3^–^5^ High spatial resolution is especially helpful in brachytherapy dosimetry in which there is a high dose gradient near the sources. They can also provide two dimensional dose distributions near these regions.^4^

The radiochromic films can be read with a number of devices such as film digitizers, micro-densitometers, and laser based scanners.^4^ Color/document scanners have also been used as a reader for radiochromic films. For example Agfa Arcus II, HP ScanJet II cx, Epson Pro 1680 Expression and some other different models of colour scanners have been evaluated for this purpose.^6^–^10^ The advantage of such scanners is their lower price when compared with other radiochromic film densitometers.

However, with these scanners, it is possible to measure the film response in one of the three major wavelengths (red, green, and blue) and one must be careful to properly calibrate these devices for measurements of dose.^11^ Commonly, the images are reduced to only the red color channel which is equivalent to using red filters.^12^

Recently, a new Microtek (ScanMaker 1000XL Pro: Microtek International Inc., Hsinchu, Taiwan) color scanner became commercially available. This scanner is a flatbed scanner with a cold cathode fluorescent lamp and a Tri-linear charge coupled device (CCD) array. Its maximum color depth is 48 bits per pixel (16 bits per each color channel). This scanner is capable to scan images of maximum 12×17 size with 3200×6400 dpi optical resolution and a maximum optical density of 4.0. Scanning may be performed either in reflection or transmission mode. Although there are reports on the application of different models of color/document scanners^13^–^14^, to our knowledge, this model of scanner has not yet been used as a RCF film reader.

The goal of this project is to investigate the application of the Microtek color scanner for dosimetric evaluation of a HDR GZP6 ^60^Co source. In these evaluations the HDR source will be utilized to expose the Gafchromic EBT films in a PMMA equivalent phantom material. The results of these experimental data will be compared with the data from the GZP6 treatment planning system and the results of Monte Carlo (MC) simulations as well as the published MC results by Naseri *et al*.^15^

## Materials and methods

### Radioactive source and tandem applicator

**FIGURE 1.**
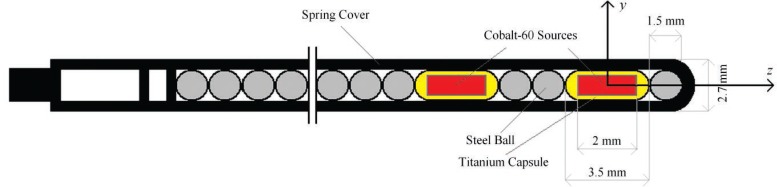
The GZP6 source braid (source number one) in the GZP6 afterloading brachytherapy unit.

GZP6 ^60^Co afterloading HDR unit (Nuclear Power Institute of China) has 6 channels with non-stepping sources in channels 1–5 and a stepping source in channel 6. In this study, dose distribution around the source in channel 1 has been evaluated. This source is composed of two active cobalt-60 pellets placed in a nickel plating encapsulation with a number of non-active spherical pellets as spacers between and outside of the active pellets (Figure 1). Each active pellet is a 2 mm long and 1 mm in diameter ^60^Co source in a Titanium capsule. The active and non active pellets are fixed in a spring cover. The straight tandem applicator (UT0°) in GZP6 is normally used for the intracavitary brachytherapy treatment of cervical cancer patients. The applicator, which is made of stainless steel, has an inner diameter of 4.3 mm, wall thickness of 0.6 mm and a body length of 25.2 cm.

### Radiochromic film calibration

Characteristics of EBT radiochromic films: energy response, dose rate dependency, uniformity, post irradiation density growth etc. have been analyzed and discussed elsewhere.^16^–^18^ In the present study, several sheets (20.3 cm × 25.4 cm) of EBT GafChromic^®^ (lot number 34351-05, International Specialty Products, Wayne, NJ, USA) film were cut into 48 pieces of 2×3 cm^2^ for irradiation. Care was taken to select the pieces from the same batch, for each measurement. Each piece was given an identity number, prior to the measurements.

The irradiated films were scanned by a Microtek ScanMaker 1000XL Pro color scanner. To obtain background optical density (OD) all 48 film pieces were scanned 24 hours prior to calibration. To minimize the warming up effect, the scanner was turned on at least 30 minutes before scanning. All film pieces were scanned by placing them at the center of the scanner bed and selecting 100 dpi resolutions. The films were scanned in 48-bit RGB color mode with the maximum OD range without applying any corrections or enhancements by the scanner’s software. Since for EBT films the response is greatest in the red color channel^16^, the red components of RGB images were selected to derive the background OD of the films. All of images were saved as uncompressed tagged image file format (TIFF) files. To reduce noise effect, each film was scanned three times in transmission mode. As it has been recommended for EBT film, they were positioned in landscape orientation on the scanner bed. In addition, consecutive scanning of the films was avoided to minimize the film heating effects that could change the OD of the films. Appropriate care was taken to avoid dust particles and scratches on the films during film storage, scanning and calibrations.

Each group of films were calibrated using a Theratron 780C cobalt unit in the center of a 20×20 cm^2^ field inside a dose range of 0.5–35 Gy inside a 32×32×31 cm^2^ water phantom. The output of the cobalt unit was measured in the same conditions using a 0.6 cm^2^ Farmer 2581 ionization chamber. The chamber reading was corrected for the ambient temperature and pressure. The films were scanned 24 hours after irradiation according to the aforementioned protocol. Net optical density (NOD) was calculated from equation [1] as the difference of averaged calibration and background optical density:

[1] $$\text{NOD}={\text{OD}}_{\text{cal}}-{\text{OD}}_{\text{back}}=-({\text{log}}_{10}(P_{\text{cal.}})-{\text{log}}_{10}(P_{\text{back}}))$$

where: OD_cal_ is optical density of the calibration film, OD_back_ is background optical density (for unexposed film), *P*_cal_ is pixel value for calibration film and *P*_back_ is pixel value for the background film. The dose (Gy) was plotted versus the NOD and an exponential function was fitted to the curve.

Image manipulations such as: extraction of red component from the RGB images, calculation of mean pixel values (for a ROI on the whole film’s surface except of the film edges), OD and dose mapping, dose contouring, etc were accomplished by means of appropriate scripts compiled accordingly in MATLAB (version 7.2.0.232, The Math Works, Inc., Natwick, MA) environment.

### In-phantom measurements

**FIGURE 2.**
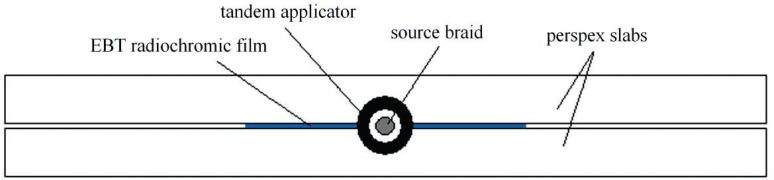
Schematic diagram showing the configuration of the EBT radiochromic film, GZP6 tandem applicator and the PMMA slabs during the film irradiation.

An EBT film sheet (from the same batch used for calibration) was cut in such a way that could house the GZP6 channel 1 applicator, the applicator tip was positioned symmetrically from the film edges. The film was scanned according to the protocol explained earlier in this section. The film was accommodated in a 50×50×50 cm^3^ cubic PMMA phantom made from 1 cm thick slabs. Figure 2 shows the configuration of film, applicator and slabs during the irradiation.

To irradiate the film, a dose of 5 Gy was delivered to the point of 2 cm above and 2 cm lateral to the source position by GZP6 afterloading system. This was equal to a period of 671.3 s irradiation time in the date of measurements. According to the previous protocol, the film was scanned 24 hours after irradiation. The net optical densities in the measurement film’s image then were correlated to the dose value by applying the calibration formula (equation [4]).

### EBT film sensitivity to the scanner’s light source

Radiochromic films are sensitive to infra red (IR) light, therefore it is recommended to protect them against IR sources and avoid multiple consecutive scanning.^19^ Radiochromic films are also sensitive to UV light.^3^ UV source may include light bulbs, fluorescent lamps, sunlight and even light from the film scanner. The films sensitivity to the scanner’s light source was examined by the following manner: (1) Nine 20.3×12.7 cm^2^ unexposed film pieces were scanned 15 times to assess whether this numbers of scanning would cause higher OD (or dose reading). (2) Mean optical density of individual film pieces in the red channel for the RGB image was obtained before and after the 15 times of scanning. This was accomplished by averaging the pixels values over the entire area of each film (except for the film edges).

### Uniformity checking

The combined effect of non-uniform response of EBT film and the Microtek scanner was examined. For this purpose nine 20.3×12.7 cm^2^ pieces of films (from the same film batch already used) were irradiated in a clinically assumed uniform field. The films were scanned by the scanner before irradiation to obtain background pixels values for each film. The films were subjected to a wide range of absorbed dose 0.5–25 Gy, delivered in a 20×20 cm^2^ 6 MV photon field of an ELEKTA linac (model SL75/25). To accomplish this protocol, films were positioned centrally at the depth of 10 cm in a solid water phantom (SP34, Wellhofer Scanditronix GmbH, Schwarzenbruck, Germany). Symmetry and flatness of the irradiation field were examined by an automated scanning water phantom system (RFA300plus: Scanditronix-Wellhofer, Nüremberg, Germany) before film irradiations. The films were scanned 48 hours following to irradiations.

The film pieces were positioned in the central part of the scanner’s bed when scanning. The dose value represented by every film pixel was determined by the calibration fitted curve on the central area of 16×11 cm^2^ size of each film. The images were saved as TIFF files and the red channel of RGB image was chosen to extract dose information. A wiener filter of 5 pixels×5 pixels was applied on the saved image of the films before image processing. Wiener filter is an adaptive filter predefined in MATLAB which is used to reduce the amount of noise present in an image. Film uniformities were evaluated in terms of coefficient of variation (in percentage) of dose for a region of interest (ROI) of 16×11 cm^2^ on each film sheet. Coefficient of variation is defined as:

[2] $$\text{CV}=\frac{100\times \sigma }{\mu }$$

where σ is standard deviation and *μ* is mean dose value. The peak-to-peak variation of optical density along longitudinal direction (the direction of scanning) and transverse direction (orthogonal to the scanning direction) of the films were also evaluated for the 9 film pieces on the central area of 16×11 cm^2^ size on each film piece:

[3] $${\Delta \text{OD}}_{\text{peak}}=\frac{{\text{OD}}_{\text{max}}-{\text{OD}}_{\text{min}}}{{\text{OD}}_{\text{central pixel}}}$$

where OD_central pixel_ is optical density value of central pixel of the film sheet in the longitudinal/transverse direction.

### Monte Carlo (MC) simulations

MCNPX version 2.4.0 MC code was used as the simulation tool in this study.^20^ The loaded applicator including applicator body, active sources and inactive pellets were simulated. The applicator was positioned at the centre of a cylindrical PMMA phantom 25 cm in radius and 50 cm in length. A mesh (14 cm×14 cm×0.05 cm consisting of 0.5 mm^3^ voxels) was used to score deposited dose, using MCNPX type 1 tally with pedep option.^21^ This tally scores the average energy deposition per unit volume (MeV/(cm^3^.source-particle)). Then the dose value assigned to each voxel was determined by multiplying the tally value by a conversion factor; including the sources activity and source particles per disintegration, irradiation duration according to in-phantom irradiation and so on. A total number of 5×10^8^ primary photon histories were simulated. The average uncertainty was 1.86% over the whole mesh voxels. Since the uncertainty on the source activity certified by the manufacturer is ±10%, the overall uncertainty of MC calculations will not be less than that.

### GZP6 treatment planning system

GZP6 afterloading unit, manufactured by Nuclear Power Institute of China (NPIC)^22^, incorporates an afterloader, a source container and a treatment planning system. The GZP6 treatment planning system uses Sievert integral to calculate dose distributions around the GZP6 sources.^23^ The GZP6 treatment planning system produces 2D dose distributions in the transverse and longitudinal planes related to the selected source (or sources) configuration.

## Results

### Radiochromic film measurements

**FIGURE 3.**
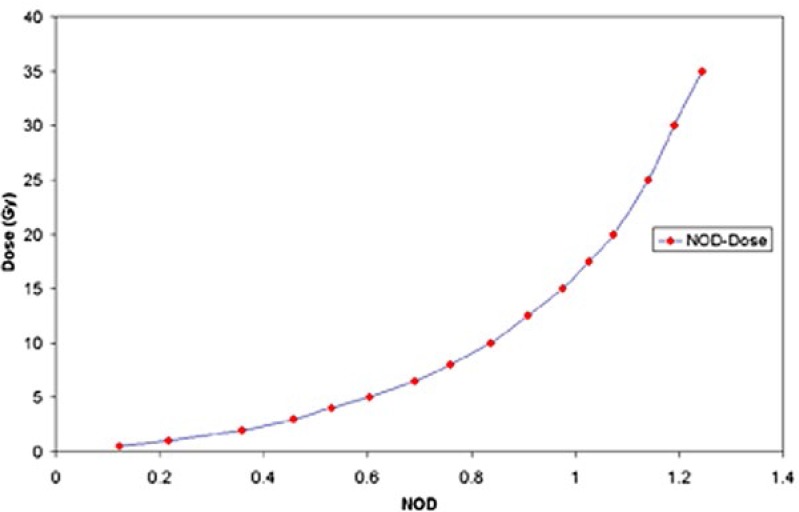
Calibration curves (dose versus net optical density (NOD)) obtained for EBT films for the red colour channel.

Figure 3 shows the calibration curve (dose versus NOD) obtained for the EBT films.

Delivered dose *D* (in Gy) versus measured NOD can be obtained from the fitted formula using the following equation:

[4] $$D=0.7424{\text{e}}^{3.094\text{NOD}}-0.8355{\text{e}}^{-4.848\text{NOD}}$$

The R-square value for the fitting is equal to 0.9995.

### EBT film sensitivity to the scanner’s light sources UV component

The results of 15 times scanning with Microtek scanner have raised to an average change of 0.00157 in optical density of the nine scanned film pieces. This value equals to a net optical density of 0.000105 per scanning. With regard to our film calibrations, a dose of 0.5 Gy equals to a net optical density of 0.122. Thus, it can be concluded that scanning with Microtek scanner does not significantly change the optical density of EBT radiochromic film, due to the light source or the UV component of the light source.

### Uniformity checking

As it is mentioned in the materials and methods section, symmetry and flatness of the irradiation field were examined by an automated scanning water phantom. The resulted symmetry and flatness were 2% and 2.8% respectively. Mean value of coefficient of variation for represented absorbed dose by the 9 film pieces was 2.99%. Mean ΔOD_peak_, averaged for 9 film pieces, in the longitudinal and transverse direction of the film were respectively 2.85% (1.59–4.06%) and 4.97% (2.47–8.50%).

### Comparisons of dose distributions

**FIGURE 4.**
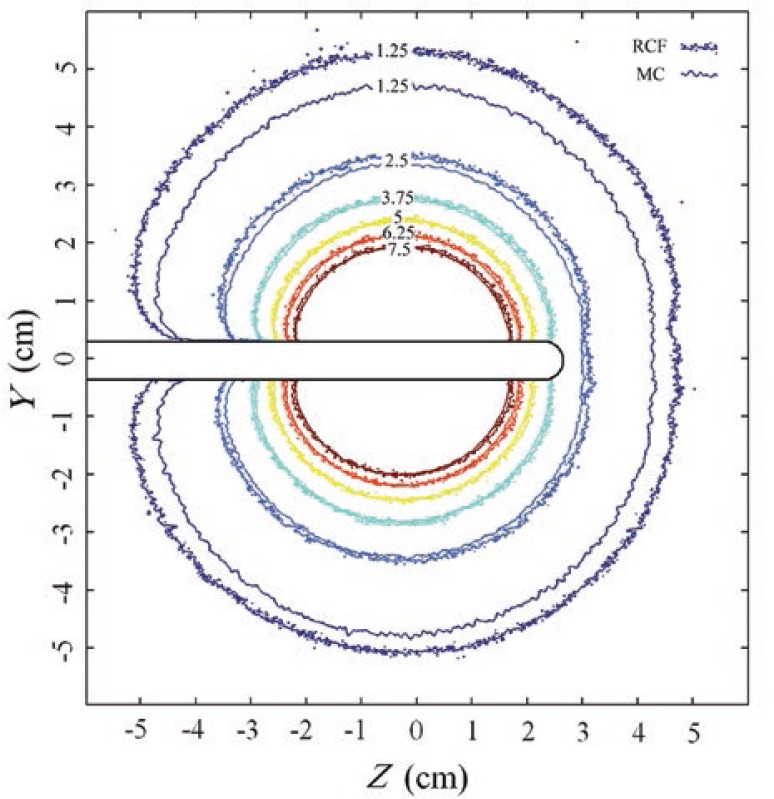
Dose distributions (in Gy) for the GZP6 source number one around the tandem applicator in a plane parallel to the long axis of the applicator as obtained by radiochromic films (RCF) measurements and Monte Carlo (MC) simulations.

Dose distributions for the GZP6 source number one around the tandem applicator, obtained by radiochromic film measurement and MC simulation are presented in Figure 4. The curves are representing isodose lines of 1.25–7.5 Gy.

Calculated and measured dose values are only different by 4% on average. The difference is higher (nearly 20%) for the dose value of 1.25 Gy.

**FIGURE 5.**
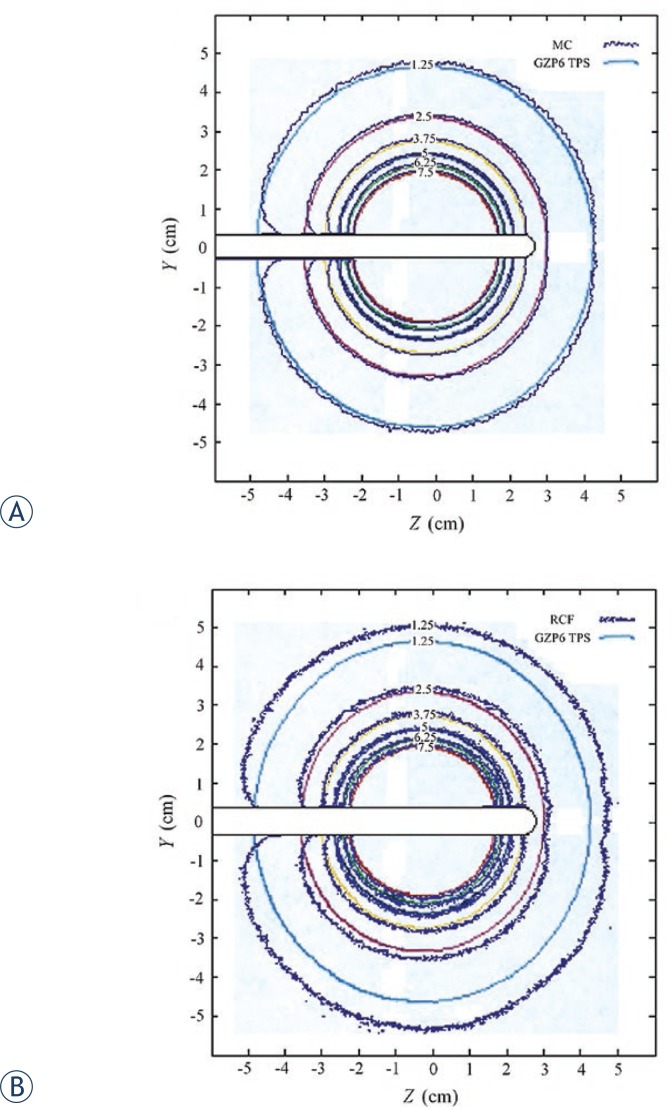
Dose distribution (in Gy) for the GZP6 source number one around the tandem applicator in a plane parallel to the long axis of the applicator (A) GZP6 treatment planning systems (TPS) versus Monte Carlo (MC) calculations (B) GZP6 TPS versus radiochromic films (RCF) measurement.

Dose distribution around the tandem applicator as provided by the GZP6 treatment planning systems (TPS) versus calculated values by MC and radiochromic film measurements are shown in Figure 5.

When calculated, an average dose difference of 4% was observed between the dose contours representing MC simulation and TPS. The difference between dose values obtained from radiochromic film measurement and those provided by TPS is in order of 5%. The difference is more pronounced for lower doses (18% for 1.25 Gy). A summary of dose distribution differences related to MC, RCF and GZP6 TPS are presented in Table 1. The Table also presents the difference between MC versus GZP6 TPS isodose lines (in percent) for the study by Naseri *et al*.^15^

## Discussion

We have used a Microtek ScanMaker 1000XL Pro scanner as a radiochromic film reader. Different features of the scanner were examined for this purpose. Scanning of uniformly irradiated EBT film pieces on ROIs of 16×11 cm^2^ size have demonstrated acceptable nonuniformity levels in both longitudinal and transverse directions. This is particularly true when compared to the study of Lynch *et al.*, in which 7% and 17% and variations in optical density for an Epson scanner and 12% and 8% for a Microtek scanner was reported respectively in the longitudinal and transverse directions.^19^ Our results of examining EBT sensitivity to UV component of the scanner’s light source has showed that multiple scanning of a film does not considerably change its optical density.

We have obtained isodose curves around a GZP6 tandem applicator first by MC simulations and then by film dosimetry by EBT radiochromic films. Generally the results are indicating there is a good agreement between radiochromic film measurement, MC simulations and GZP6 TPS. However the differences between the radiochromic film-MC, and radiochromic film-TPS for the dose contour of 1.25 Gy are 20% and 18% respectively. This implies that for dose values of equal to or less than 1.25 Gy the noise contribution of the scanner is high, giving rise to a high error.^21^ The deviation could be attributed to our fitting curve to the calibration data, so that it has a deviation of 15.7% for the dose of 1 Gy.

By taking into account our MC uncertainty, it can be concluded that the difference between MC calculation versus the GZP6 TPS and radiochromic film measured dose distributions are within the uncertainties expected for MC calculations and radiochromic film measurements. Compared to our study, the less difference between MC calculations and GZP6 treatment planning system in the study of Naseri *et al.*^15^ can be related to the fact that in that study there were a comparison between the relative isodose contours (in percent) but in this study the isodose curves were compared in terms of Gy. There is also a minor difference between the types of applicators simulated in two studies.

As it is evident from Figure 5, there are also some attenuation effects near the tip and base of the applicator which is not taken into account by the GZP6 planning system while it is considered by the radiochromic film measurement and MC calculations.

A rigorous scanning protocol was employed for film scanning. The protocol includes: avoiding multiple consecutive scanning; films were scanned always in landscape orientation; images were saved as uncompressed TIFF files; they were handled in a relatively stable temperature; noise effects were eliminated by pixel averaging of multiple scanning of film pieces in different steps of reading. It is concluded that by using a standard protocol, the Microtek ScanMaker 1000XL Pro can be used as a radiochromic film reader in combination with EBT radiochromic film. The combination can be used as a practical tool for HDR brachytherapy dose measurements. Since a color scanner has not appropriate software for reading RC films, designing appropriate MATLAB functions is necessary in the different steps of film reading; for example to extract pixel values, film calibration and correlation of pixel values to dose values on the RCF images.

## Figures and Tables

**TABLE 1.** Dose differences between MC, RCF and TPS dose distributions in this study (for dose contours of 2.5–7.5 Gy) and that by Naseri *et al*^15^ (for dose contours 10–250%).

	**This study**		**Naseri*et al***
RCF-MC	MC-GZP6 TPS	RCF-GZP6 TPS	MC-GZP6 TPS
4%	4%	5%	<2%
